# Resveratrol-Loaded Polymeric Nanoparticles: The Effects of D-α-Tocopheryl Polyethylene Glycol 1000 Succinate (TPGS) on Physicochemical and Biological Properties against Breast Cancer In Vitro and In Vivo

**DOI:** 10.3390/cancers15102802

**Published:** 2023-05-17

**Authors:** Paulo George Cavalcante de Freitas, Bruno Rodrigues Arruda, Maria Gabriela Araújo Mendes, João Vito Barroso de Freitas, Mateus Edson da Silva, Tiago Lima Sampaio, Raquel Petrilli, Josimar O. Eloy

**Affiliations:** 1 Department of Pharmacy, Faculty of Pharmacy, Dentistry and Nursing, Federal University of Ceará, Fortaleza 60430-160, CE, Brazilmateusedson20@gmail.com (M.E.d.S.); tiagosampaio@ufc.br (T.L.S.); 2 Department of Physiology and Pharmacology, Faculty of Medicine, Federal University of Ceará, Fortaleza 60430-160, CE, Brazil; 3 Institute of Health Sciences, University of International Integration of the Afro-Brazilian Lusophony—UNILAB, Redenção 62790-000, CE, Brazil; 4 Pharmaceutical Sciences Graduate Course, Federal University of Ceará, Fortaleza 60430-160, CE, Brazil

**Keywords:** resveratrol, TPGS, nanoparticle, poly-ε-caprolactone, breast cancer

## Abstract

**Simple Summary:**

Resveratrol is a compound that has demonstrated anti-proliferative effects on several cancer cell lines. However, resveratrol is a lipophilic drug and its therapeutic effect can be improved by encapsulation in polymeric nanoparticles. Furthermore, functionalization of polycaprolactone-based polymeric nanoparticles with the non-ionic surfactant TPGS was reported to reduce drug resistance. Thus, this study aimed to develop nanoparticles loaded with resveratrol and investigate the effect of TPGS on the physicochemical characteristics of nanoparticles and their biological effects, in vitro and in vivo, in a breast cancer cell line. The nanoparticles had a size of 138.6 nm and encapsulation efficiency of 96.6%. Cytotoxicity tests indicated potentiation of the cytotoxic effect of resveratrol when encapsulated, and flow cytometry and confocal microscopy tests indicated excellent cell uptake dependent on the concentration of nanoparticles, suggesting that TPGS may represent a problem in nanoparticle endocytosis. The in vivo study evaluating the antitumor activity of the nanoparticles confirmed the data obtained in the in vitro tests. In addition, the biochemical evaluation showed possible hepatotoxicity for the formulation with TPGS.

**Abstract:**

Resveratrol (RSV), a phytoalexin from grapes and peanuts, has been reported to exhibit antiproliferative effects on various cancer cell lines. In breast cancer, RSV has been demonstrated to exert an antiproliferative effect on both hormone-dependent and hormone-independent breast cancer cell lines. However, RSV is a lipophilic drug, and its therapeutic effect could be improved through nanoencapsulation. Functionalizing polymeric nanoparticles based on polycaprolactone (PCL) with polyethylene glycol 1000 tocopheryl succinate (TPGS) has been reported to prolong drug circulation and reduce drug resistance. However, the effect of TPGS on the physicochemical properties and biological effects of breast cancer cells remains unclear. Therefore, this study aimed to develop RSV-loaded PCL nanoparticles using nanoprecipitation and investigate the effect of TPGS on the nanoparticles’ physicochemical characteristics (particle size, zeta potential, encapsulation efficiency, morphology, and release rate) and biological effects on the 4T1 breast cancer cell line (cytotoxicity and cell uptake), in vitro and in vivo. The optimized nanoparticles without TPGS had a size of 138.1 ± 1.8 nm, a polydispersity index (PDI) of 0.182 ± 0.01, a zeta potential of −2.42 ± 0.56 mV, and an encapsulation efficiency of 98.2 ± 0.87%, while nanoparticles with TPGS had a size of 127.5 ± 3.11 nm, PDI of 0.186 ± 0.01, zeta potential of −2.91 ± 0.90 mV, and an encapsulation efficiency of 98.40 ± 0.004%. Scanning electron microscopy revealed spherical nanoparticles with low aggregation tendency. Differential Scanning Calorimetry (DSC) and Fourier Transform Infrared Spectroscopy (FTIR) identified the constituents of the nanoparticles and the presence of drug encapsulation in an amorphous state. In vitro release studies showed that both formulations followed the same dissolution profiles, with no statistical differences. In cytotoxicity tests, IC_50_ values of 0.12 µM, 0.73 µM, and 4.06 µM were found for the formulation without TPGS, with TPGS, and pure drug, respectively, indicating the potentiation of the cytotoxic effect of resveratrol when encapsulated. Flow cytometry and confocal microscopy tests indicated excellent cellular uptake dependent on the concentration of nanoparticles, with a significant difference between the two formulations, suggesting that TPGS may pose a problem in the endocytosis of nanoparticles. The in vivo study evaluating the antitumor activity of the nanoparticles confirmed the data obtained in the in vitro tests, demonstrating that the nanoparticle without TPGS significantly reduced tumor volume, tumor mass, maintained body weight, and improved survival in mice. Moreover, the biochemical evaluation evidenced possible hepatotoxicity for formulation with TPGS.

## 1. Introduction

Breast cancer is considered the leading cause of death worldwide among women [1]. The treatments available depend on the characteristics of the cancer (tumor size, degree of tumor differentiation, presence of specific receptors) and the patients (obesity, diet, age). A molecular target studied is the epidermal growth factor 2 (HER2) which is also found with abnormal expression in 20% of breast cancers [2]. An example of targeted therapy is the use of the monoclonal antibody Trastuzumab^®^, which acts on HER2-positive breast cancer [3]. There are several drugs used in the treatment of breast cancer, however, they have important disadvantages, such as low bioavailability and non-specific distribution, which leads to a range of side effects. In addition, they have low solubility in water which leads to low bioavailability. Thus, considering the growth estimation in the number of cases in the near future, the search for new forms of treatment is extremely important.

Resveratrol (RSV) is a lipophilic molecule with a water solubility of only 3 mg/100 mL [4]. Although it has low solubility in water, this molecule has excellent membrane permeability due to its high lipophilicity [5]. Studies show the therapeutic action of this drug against several types of cancer, including breast cancer, where its inhibitory action was observed in several different cancer cell lines, being effective both in those that have receptors for estrogen and progesterone and in those that do not express these receptors. This molecule also shows positive results in HER2 positive and negative cell lines, with dose-dependent inhibition directly proportional to the exposure time. Such responses are due to the action of the drug on several targets that are not fully understood, but some of these mechanisms are the inhibition of the ER/protein kinase B receptor; inhibition of IGF-2 precursors (insulin-like growth factor) which, when suppressed, leads to cell inhibition [5,6]. However, this drug has limited bioavailability, as its metabolization occurs quickly, preventing much of the free form from reaching the site of action [7]. In this case, the encapsulation of this molecule by nanoparticles is considered an interesting alternative, as it allows a more effective drug action.

The field of pharmaceutical nanotechnology allows countless possibilities for improving formulations as it increases their effectiveness, associated with the reduction of their side effects. Polymeric nanoparticles (NPs) have greater biocompatibility than other materials, have low or no toxicity, and can be biodegradable. NPs can be made from natural or synthetic polymers, the former is generally abundant in nature and inexpensive, in addition to presenting high biocompatibility and low immunogenicity. However, NPs have disadvantages such as greater variation in characteristics from batch to batch, lower purity, and greater structural complexity [8,9]. The management of superficial characteristics of NPs is considered an obstacle, as these are directly related to the susceptibility of NPs to be recognized and eliminated by the immune system.

In this sense, D-α-tocopheryl polyethylene glycol 1000 succinate (TPGS) stands out, which is a surfactant that helps NPs to improve the release of the encapsulated drug and increase encapsulation efficiency [10,11,12]. Studies show that in addition to improving pharmacokinetic and physicochemical characteristics, this surfactant intrinsically has a relevant antitumor action, as it inhibits the action of P-glycoprotein [12]. TPGS acts synergistically with other antitumor drugs, as it has the ability to cause cancer cell apoptosis, and such ability also correlates with its action on mitochondria. In addition to acting via the mitochondrial complex, TPGS can also inhibit the cell cycle, which will allow the activation of pro-apoptotic genes that lead to cell death [10,11,13]. Another prominent polymer is poly-ε-caprolactone (PCL), which was approved by the Food and Drug Administration (FDA) for use in the production of drugs and surgical materials [14]. This polymer has physicochemical characteristics that make it an excellent material for the production of NPs for drug delivery. It has high versatility that comes from the high number of changes that can be made in its chemical, physical, and mechanical characteristics, which reflect on the crystallinity, solubility, and ionic characteristics, among others.

Although RSV has already been encapsulated in polymeric nanoparticles [15], the role of TPGS on both the physicochemical properties of nanoparticles and the biological behavior of breast cancer remains unclear. Thus, the purpose of this work is the development and characterization of NPs based on PCL for RSV encapsulation in addition to observing the influence of TPGS on the physicochemical and biological properties of NPs, with the objective of generating a potentially applicable formulation for the treatment of cancer. In this sense, NPs based on PCL and TPGS containing RSV were developed, as well as thoroughly characterized for their physicochemical properties, and evaluated for cytotoxicity, cellular uptake, and in vivo anticancer activity in an animal model of breast cancer.

## 2. Material and Methods

### 2.1. Materials

Resveratrol (RSV) was supplied by Suzhou Vitajoy (Suzhou, China). D-α-tocopheryl polyethylene glycol 1000 succinate (TPGS) was obtained from BASF Chemicals (Mississauga, ON, Canada). Trypsin (0.25%), fetal bovine serum, dimethyl sulfoxide (DMSO), 3,3′-dioctadecyloxacarbocyanine perchlorate (DiO), 3-[4,5-dimethyl-thiazol-2-yl]-2,5-diphenyltetrazolium bromide (MTT), poly-ε-Caprolactone (PCL) flakes or chunks with an average Mw ~14,000, were purchased from Sigma Aldrich Co. (St. Louis, MO, USA). Propidium iodide was purchased from Thermo Fisher Scientific (Waltham, MA, USA). Precision Plus Dual Color Protein was obtained from BioRad (Hercules, CA, USA). Poloxamer 407 (Synperonic™ PE/F68 pharma) was a kind gift from CRODA (Campinas, SP, Brazil). Sodium chloride, potassium chloride, and potassium phosphate monobasic were purchased from Merck (Rahway, NJ, USA). Solvents acetone and acetonitrile were obtained from Merck and J.T. Baker^®^ (Phillipsburg, NJ, USA), respectively. Cellulose dialysis membranes (12–14 kDa MWCO (molecular weight cut-off)) were obtained from Fisherbrand (Loughborough, Leics, UK).

### 2.2. Preparation of PCL-Based Nanoparticles with and without TPGS

The preparation of polymeric nanoparticles was performed using the nanoprecipitation technique [16]. The acetone-based organic phase was tested in two different volumes, either 5 mL or 10 mL, in which RSV was solubilized in different amounts, 1, 5, or 10 mg. In this same phase, 10 mg of PCL polymer flakes were also dissolved. The aqueous phase (20 mL) was composed of both 0.15% (*w*/*v*) of Poloxamer 407 with or without TPGS solubilized in different ratios 0.005%; 0.01%; 0.015%; 0.03% and 0.05% (*w*/*w*) in relation to the amount of PCL in phosphate-buffered saline (PBS), pH 7.4. The next step of the technique was the slow dripping of the organic phase into the aqueous phase, which was constantly stirred at 200 rpm at 30 °C, overnight [17].

For the solid-state characterization of the nanoparticles and for the stability study, they were lyophilized using ratios of 10%, 20%, and 30% of sucrose as a cryoprotectant, in relation to the amount of PCL. Samples were frozen at −80 °C for 24 h and then lyophilized for 48 h (FreeZone 4.5 Liter Freeze Dry Systems-Labconco). Lyophilized samples were stored in sealed falcon tubes and conditioned in a refrigerator.

### 2.3. Physicochemical Characterization of PCL-Based Nanoparticles with and without TPGS

#### 2.3.1. Particle Size, Polydispersity Index (PDI), and Zeta Potential

The hydrodynamic size distribution, DI, and zeta potential of the NPs were determined at 25 °C by dynamic light scattering measurements, using HeNe laser operating at 4 mW and 633 nm wavelength at an angle of 90°, in a Malvern Nanosizer ZS equipment (Malvern Instruments, Malvern, UK). Samples were diluted (1:10) in ultrapure water and vortex mixed before analyses. All measurements were performed in triplicate and results were expressed as mean ± standard deviation (SD).

#### 2.3.2. Gas Chromatography with Flame Ionization Detector

The analysis of the residual acetone content in the formulations was performed by gas chromatography with a flame ionization detector (GC-FID, Young Lin Instrument (Anyang, Republic of Korea), model YL6100), with split-mode injection system (1:20 ratio) and 0.25 mm (internal diameter) × 30 m (L) fused-silica column coated with a 0.25 µm film thickness 5% phenyl–95% dimethylpolysiloxane from Zebron. Nitrogen was the carrier gas with a 4 mL/min of flow rate. The method program was as follows: initially, the temperature was maintained at 38 °C for three minutes. Then increased by 1 °C/min until reaching 40 °C, followed by an increase of 10 °C/min until reaching 120 °C. Injector and detector temperatures were maintained at 250 °C and 300 °C, respectively. Sample solutions of formulations diluted in ultra-purified water (1:10, *v*/*v*) were injected (1 µL) and acetone content was calculated based on the acetone standard curve (0.5–10%).

#### 2.3.3. Encapsulation Efficiency

RSV encapsulation efficiency was determined by the ultrafiltration method described by Mussi et al. (2013), with some modifications. For this, 1 mL of the formulations was transferred to a 10 mL volumetric flask, which was completed with acetonitrile, in order to disrupt the nanoparticles. The flask was vortexed, subjected to an ultrasonic bath for 10 min, and then the contents were filtered through a 0.45 µm polytetrafluoroethylene (PTFE) filter for total drug quantification. For the quantification of the free drug sample, a new aliquot of 2.5 mL of the formulations was removed and filtered and then transferred to a 50 KDa Amicon tube. The Amicon tube was then centrifuged for 20 min at 4500 rpm and 1 mL of this filtrate was transferred to a 10 mL volumetric flask and completed with acetonitrile, followed by vortexing, and finally, the solution was filtered through a 0.45 µm PTFE filter [18]. The assay was performed in triplicate. Afterward, the sample’s absorbance was determined by spectrophotometry at a wavelength of 325 nm, and drug concentration was calculated from the calibration curve. The encapsulation efficiency (EE%) was calculated according to Equation (1):

(1) $$EE\left(\%\right)=\frac{Total\;amount\;of\;Resveratrol-amount\;ofultrafiltered\;resveratrol}{Total\;amount\;ofResveratrol}\times 100$$

#### 2.3.4. Stability of Polymeric Nanoparticles

The stability of both liquid and lyophilized formulations was monitored at 4 °C for 0, 7, 14, 30, 60, and 90 and characterized by particle size, PDI, and zeta potential [19].

#### 2.3.5. Morphology of Nanoparticles

The morphology of the NPs was examined using scanning electron microscopy (SEM) [20]. The samples were placed on an aluminum sample mounter, on a carbon tape, dried at room temperature, and spray-coated with gold, in order to increase the surface conductivity. The analysis was performed at an accelerating voltage of 10 kV in a scanning electron microscope, the Quanta 450 FEG—FEI, with a nominal resolution of 1 nm.

#### 2.3.6. Thermal Analysis

Differential Scanning Calorimetry (DSC) curves of free RSV and NPs containing RSV were obtained in the calorimeter of the DSC-60 Plus series Shimadzu. The samples were placed in aluminum crucibles with lids and heated from 30 °C to 350 °C at a rate of 10 °C/min, under a nitrogen pressure of 3 kgf/cm^2^, for the characterization of endothermic and exothermic events [20].

#### 2.3.7. Fourier Transform Infrared Spectroscopy (FTIR)

For the FTIR analysis, the lyophilized nanoparticles were mixed with potassium bromide and compressed in a hydraulic press. Scanning was performed at 4 cm^−1^ resolution, from 400 to 4000 cm^−1^ on the Varian spectrometer, model FT-IR Spectrum 660-IR [21].

#### 2.3.8. Determination of the RSV In Vitro Release Profile

For the quantification of RSV release from nanoparticles, a High-Performance Liquid Chromatography (HPLC) method was used. The chromatograph used was a Hitachi^®^ LACHRON ULTRA, using a SunFire C18 column (3.5 µm, 4.6 mm × 150 mm). The chromatographic conditions followed a previous work with modifications [22]. The method employed an isocratic mobile phase composed of acetonitrile: water (60:40 *v*/*v*), a volume injection volume of 20 μL, a flow rate of 0.75 mL·min^−1^, an oven temperature of 25 °C, and a wavelength at 306 nm. The calibration curve was made from a 1 mg/mL resveratrol stock solution. From this solution, five curve points were prepared, namely: 0.5 µg/mL; 1.0 µg/mL; 2.0 µg/mL; 15.0 µg/mL; 30.0 µg/mL, diluted in acetonitrile and filtered through PTFE [22].

The release of RSV was investigated using the cellulose dialysis membrane method, using an Erweka dissolutor, model DT 80, with agitation at 150 rpm, at 37 °C. In this study, samples were dispersed in 1 mL of PBS buffer, pH 7.4, with 1% sodium lauryl sulfate, and placed inside polyvinyl chloride (PVC) tubes wrapped with 12–14 kDa MWCO (molecular weight cut-off) cellulose dialysis membranes and connected to the dissolution shafts of the apparatus. At defined interval points until 48 h, a 1mL sample was collected from the recipient compartment, with the replacement of fresh medium. Samples were diluted in acetonitrile and analyzed by HPLC using the method previously described [22]. In the end, the data were applied to the DDSolver tool (Microsoft Excel^®^ Extension) for statistics, mathematical analysis, and kinetic order of release through models: zero order (Dt = D0 + K0t), first order (log C = log C0 − Kt/2.303), Peppas (Mt/M∞ = Kt n), and Higuchi (ft = Qt = √D (2C − Cs)Ct) [23,24].

### 2.4. Cell Studies

#### 2.4.1. Cellular Viability Assay

The cytotoxicity assay was carried out in murine mammary carcinoma cell line, 4T1 (ATCC CRL-2539), cultured in RPMI 1640 medium, supplemented with 10% fetal bovine serum (FBS) and 1% antibiotic/antimycotic solution, at 37 °C with 5% CO_2_, according to recommendations from the American Type Culture Collection (ATCC). Only after reaching 90% confluence the cells were trypsinized and transferred to a 96-well flat bottom plate (1 × 10^4^ cells per well) and incubated for 24 h at 37 °C. Once incubated, the medium was removed, the wells washed with saline solution, and the positive and negative controls were added, free RSV, from a stock solution prepared in DMSO 1000 µM, diluted to concentrations of 0.001 µM; 0.01 µM; 0.1 µM; 0.25 µM; 0.5 µM; 0.75 µM and 10 µM. Furthermore, RSV-loaded nanoparticles with or without TPGS were studied, as well as blank nanoparticles as control RSV encapsulated in nanoparticles with or without TPGS and nanoparticles without RSV. The plates with the experimental groups were incubated for 72 h at 37 °C [25]. After 72 h, the wells were emptied, washed with saline solution and a solution of 3-[4,5-dimethyl-thiazol-2-yl]-2,5-diphenyltetrazolium bromide—MTT (2.5 mg mL^−1^) in the incomplete medium was added to the wells which were again incubated for 4 h. At the end of the process, the solution containing the MTT present in the wells was discarded and DMSO was added, followed by homogenization to dissolve the formazan crystals. The plates were read at 570 nm, and from this, the IC_50_ was calculated (concentration necessary for the death of 50% of the cells) that takes into account the concentration-effect curve, considering the optical density of the negative control as 100%. Student’s *t*-test (*p* < 0.05) was used in statistical studies.

#### 2.4.2. Cell Uptake

##### Confocal Microscopy

Nanoparticles containing 3,3′-dioctadecyloxacarbocyanine perchlorate (DIO) were used to obtain cell uptake images in the 4T1 cell line. Thus, in a 6-well plate, sterilized coverslips were placed and over these, the medium containing the cells was added for their adhesion on the coverslip (5 × 10^5^ cells per well) incubated for 24 h. After cell adhesion, the complete culture medium was removed to apply the treatment with 2 mL of the formulations containing the fluorescent agent, and the plates were incubated for 24 h at 37 °C with an atmosphere of 5% CO_2_. Following the protocol, after incubating, the wells were washed with 2 mL of PBS, and then the cells were fixed with 1 mL of 2% paraformaldehyde solution (*w*/*v*) for 10 min, after subsequent washing with PBS and the addition of 30 μL of a 4′,6-diamidino-2-phenylindole (DAPI) solution (0.3 μg/mL) for nucleus labeling. The coverslips were washed again and, in order to maintain fluorescence, they were placed on a histological slide containing Fluoromount^®^ mounting medium. The mounted slides were then analyzed in a Zeiss fluorescence confocal microscope, LSM 710 at 40 times magnification and the wavelengths used were 488 nm and 530 nm for DIO excitation and emission, respectively. For the DAPI, the wavelengths of 405 nm/457 nm were used for excitation and emission, respectively [26].

##### Flow Cytometry

Initially, cells were transferred to a 6-well plate with a flat bottom (5 × 10^5^ cells per well) and incubated for 24 h at 37 °C in an atmosphere of 5% CO_2_ to form a monolayer of cells adhered to the bottom of the well. Once the monolayer was formed, the washing process was carried out and the formulations with nanoparticles containing the fluorescent agent DIO were added, the plate was again incubated for 24 h. After the washing, cells were trypsinized. The solution containing the suspended cells was centrifuged and the pellet was resuspended in 1 mL PBS. Afterward, 2 μL of propidium iodide (50 μg/mL) was added to each sample for analysis in a BD FACSCalibur flow cytometer with wavelengths of 488 nm for DIO excitation and 530 nm for emission. For propidium iodide, excitation and emission wavelengths of 488 and 670 nm were used, respectively [26].

#### 2.4.3. In Vivo Studies

The in vivo test aims to compare the therapeutic action between the NP04, NP15 formulations, and the free drug against the 4T1 breast cancer cell line, for which 42 female Balb/c mice were obtained with an initial weight of 20 g. The animal study was approved by the Federal University of Ceará Institutional Animal Care and Use Committee (protocol number No. 5367011220) in accordance with the National Institutes of Health (NIH) Guidelines for the Care and Use of Laboratory Animals. The animals were kept in a room with controlled temperature (25 °C) and humidity, exposed to 12 h light/dark cycles, and provided food/water ad libitum. Intramammary tumors were then developed by inoculating murine breast cancer cells (4T1), in the amount of 5 × 10^4^, in the left axillary area. The 42 animals were divided into 7 groups (n = 6): (I) Negative control (saline treatment); (II) Docetaxel; (III) RSV solution; (IV) Blank NP04; (V) NP04 RSV; (VI) Blank NP15 and (VII) NP15 RSV group [23].

After the injection, the time for the appearance of tumors was monitored until tumor volume reached 100 mm^3^, from which time the treatment started [27,28]. The administered doses of resveratrol were 10 mg/kg, every 2 days, for 14 days for both free drugs or nanoparticles [28] and the doses of docetaxel of 15 mg/kg [29,30].

Tumor dimensions were measured every two days using a digital caliper and tumor volume (mm^3^) was calculated using the tumor diameter, according to Equation (2), where d (mm) and D (mm) refer to the smaller and larger diameter of the tumor, respectively. The animals were also weighed using an analytical balance before each treatment and at the end of the experiment.

(2) $$V=d^{2}\times \frac{D}{2}$$

Sixteen days after the beginning of the treatment, the animals were sacrificed, and the tumors were removed, photographed, and weighed. Blood samples were also collected by venipuncture and centrifuged at 1500× *g* in order to obtain serum for biochemical analysis. The analyses of serum albumin, total bilirubin, and fractions and activity of the enzymes alanine aminotransferase (ALT), aspartate aminotransferase (AST), and gamma-glutamyl transferase (GGT) were performed through colorimetric assays using commercial kits (Labtest^®^, Lagoa Santa, MG, Brazil). The results of the reactions were measured using the automatic spectrophotometric analyzer Mindray BS200 (Starlab, Lauro de Freitas, BA, Brazil).

#### 2.4.4. Statistical Analysis

Student’s *t*-test (*p* < 0.05) was used in statistical studies to analyze cell viability. The result of cell internalization by cytometry was analyzed by a two-way ANOVA test with a Bonferroni post-hoc test between samples. A *p* < 0.01 was considered statistically significant. Biochemical results were statistically evaluated using post-hoc one-way ANOVA with Bonferroni as well as the in vivo tests. For all analyses, a significance criterion was set at *p* < 0.05.

## 3. Results and Discussion

### 3.1. Physicochemical Analyses

#### 3.1.1. Development and Physicochemical Characterization of PCL-Based Nanoparticles with and without TPGS

**Figure 1:**
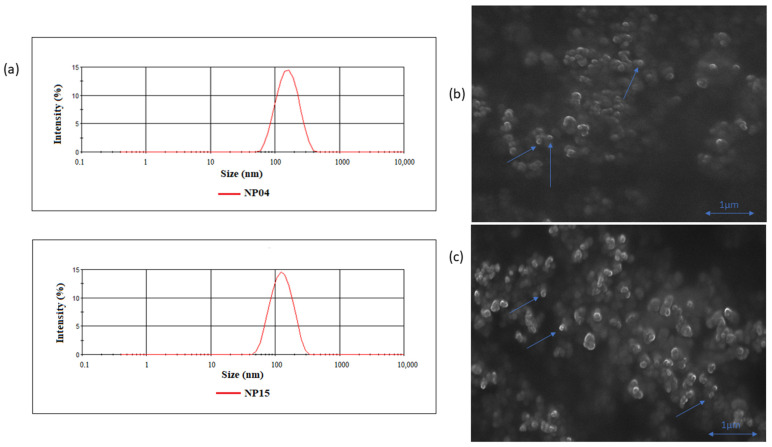
Size dispersion plots of NP04 and NP15 formulations SEM images. (**a**) Classic Gaussian curve expected in a dispersion plot indicating a single group of nanoparticles with means size around 100 nm. (**b**,**c**) SEM images of NP15 and NP04 formulations, respectively, showing spheric NPs (indicated with arrows).

The nanoparticles were successfully synthesized in the present study (Table 1). Particle size was characterized by dynamic light scattering and corroborated by SEM. Both RSV-NP and TPGS-RSV-NP present a spherical shape and mean diameter of 145 nm and 129.5 nm, respectively, as shown in Figure 1a–c. In a previous study, PCL-based NPs containing RSV with similar morphology and physicochemical characteristics were produced [15]. Such findings indicate that the method of synthesis used is robust and capable to produce nanoparticles with a size lower than 200 nm, which is of great importance when considering nanocarriers delivery to solid tumors since nanoparticles within that size range can take advantage of the enhanced permeability and retention (EPR) effect [31].

Among the formulations prepared, a narrow range of variation of physicochemical characteristics was noted, such as the PDI which remained low, and the high encapsulation efficiency in all samples with an average of 96.6%, indicating the robust encapsulation in accordance with other reports [8,21,32]. In the nanoprecipitation method, the rapid diffusion leads to the formation of the NPs. Therefore, the smaller the amount of solvent, the faster the diffusion, resulting in smaller nanoparticles. However, this does not seem to be applicable to the formulations with TPGS, which presents a smaller size in the presence of a greater quantity of solvent, which could be attributed to the presence of the two surfactants, poloxamer 407 and TPGS, that would lead to an even more accentuated decrease in the superficial tension of the aqueous phase, leading to smaller NPs [17,33].

The zeta potential varied from negative to neutral values, which is interesting, considering that a positive charge on the NPs leads to easy opsonization by the complement system, thus increasing phagocytosis. Furthermore, NPs with a neutral charge or weak negative charge possess a longer half-life in circulation, reaching easily and in more quantity than the target [34].

Among the formulations tested, the ones that presented the best results were NP04 without TPGS and NP15, containing TPGS. These formulations were analyzed by gas chromatography to determine the residual acetone content, which showed values of 0.11% ± 0.003 for NP04 and 0.12% ± 0.008 for NP15. According to the International Council for Harmonization of Technical Requirements for Pharmaceuticals for Human Use (ICH Q3C-R8) [35] and by General Method <467> Residual Solvents of the United States Pharmacopeia (USP-NF) [36], the limit for Class 3 solvents, including acetone, cannot exceed 0.5%, therefore both formulations did not exceed the limit. The presence of residual organic solvents increases the toxicity of the formulation and, in addition, can affect its physicochemical properties [37].

The lyophilization of the nanoparticles for both solid-state characterization and improved colloidal stability was carried out using sucrose, employing three different ratios, 10, 20, and 30% of sucrose to PCL polymer (*w*/*w*). After the lyophilization process of the NP04 and NP15 formulations, the physicochemical characterization was carried out. The best results were achieved with 20% sucrose, showing an average size for both NP04 and NP15 formulations equal to 185 and 150.9 nm, with a PDI of 0.396 and 0.355, respectively [38].

**Figure 2:**
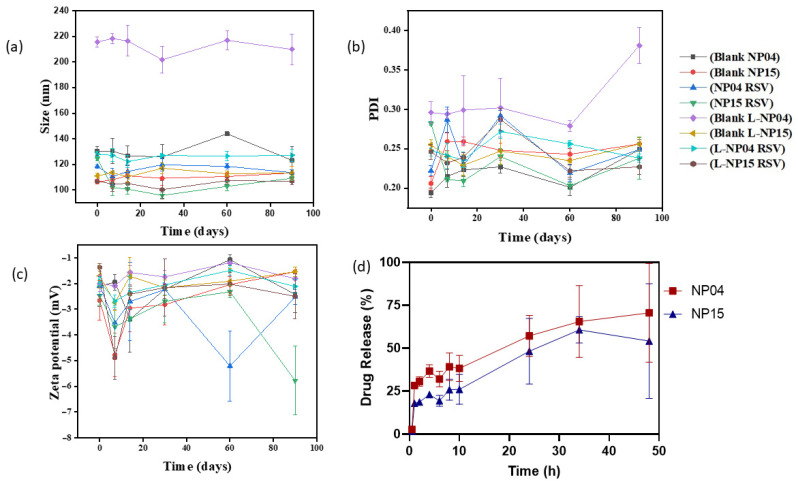
Stability of formulations for 90 days regarding particle size (**a**), PDI (**b**), and Zeta potential (**c**) for RSV-loaded NPs NP04 and NP15 and unloaded NPs after post-lyophilization (L-NP). (**d**) In vitro release assay of RSV-loaded formulations NP04 and NP15 at pH 7.4.

Figure 2 shows the data obtained by the stability test comparing the lyophilized and non-lyophilized formulations for 90 days. It is interesting to consider that there is no significant change between the lyophilized and non-lyophilized forms, indicating that stability is maintained for a long period when kept under refrigeration, a hypothesis corroborated by the PDI data, which remained constant throughout the period tested, showing that the presence of the surfactants is extremely important to maintain stability since such satisfactory results were not found in other stability tests with the same polymer [19,39].

**Figure 3:**
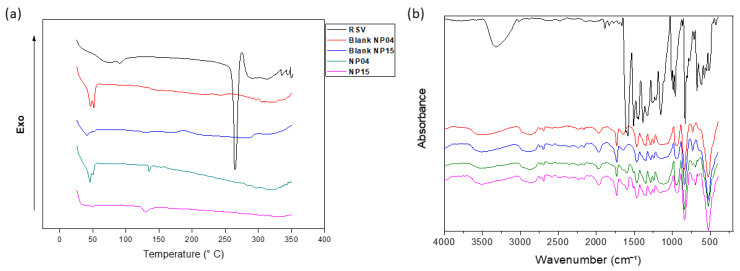
(**a**) DSC curves of free RSV, blank NP04, blank-NP15, RSV-loaded NP04, and RSV-loaded NP15. (**b**) FT-IR spectra of free RSV, blank NP04, blank-NP15, RSV-loaded NP04, and RSV-loaded NP15.

In order to investigate the physical state of RSV after encapsulation, a DSC assay was carried out. In Figure 3, an endothermic peak at 269 °C is observed, which is directly related to the melting of the crystalline RSV. In the curves referring to nanoparticles, it is possible to observe a peak at 51 °C that is related to the melting of the semicrystalline PCL polymer, which has a melting point of around 58 °C [20]. It is interesting to note that in both formulations, there are decreases in the initial value of the PCL melting point to 52.83 °C and 53.9 °C for the formulations with and without the drug, whereas, in the NP15 formulation, the PCL melting point drops to 51.17 °C in the formulation with RSV and 46.69 °C in the blank nanoparticles. Such changes are indicative of the interactions of the components of the formulation [32]. The absence of the peak referring to RSV, in addition to indicating the interaction with the components, also indicates a probable change of the drug from the crystalline state to the amorphous state [26,40].

Figure 3 shows the spectrum and all the characteristic bands for the identification of RSV, for example, the olefinic band at 1010–968 cm^−1^ and an absorption band at 1147 cm^−1^ related to the elongation C–O at 1581 cm^−1^ and 1467 cm^−1^. In addition, there is an elongation linked to C=C due to the aromatic ring present in the structure of this drug and there is also a trans olefinic band at 963 cm^−1^, in addition to the band of absorption due to the O–H stretching of the alcoholic group around 3250 cm^−1^ [21]. In the nanoformulations, it is also possible to identify bands that allow the identification of PCL, such as the axial deformation of the carbonyl at 1725 cm^−1^, a band at 2942 cm^−1^ to 2844 cm^−1^ referring to CH_2_, a band at 1292 cm^−1^ referring to C–C elongation, and the band at 1237 cm^−1^ referring to asymmetrical C–O–C stretching. The almost complete absence of RSV bands in the formulations is indicative of complete nanoparticle encapsulation, as noted by Abriata et al. (2019) in their work with PCL-based NP for paclitaxel encapsulation [21,22,26,41].

#### 3.1.2. Determination of the In Vitro RSV Release Profile

The release profile of the NP04 and NP15 formulations were studied in order to mimic the physiological pH of plasma (7.4). Among the two tested formulations, a greater release of the NP04 formulation was observed compared to NP15, as shown in Figure 2d, equivalent to 70.56%, and 54.1%, respectively, until the end of the study. Similar values have already been found for NPs based on PCL and for TPGS-based NPs. The most likely explanation for the difference in the percentages of release existing between the two formulations is the presence of TPGS, a PEGylated derivative of vitamin E, capable of acting as a hydrophilic barrier that would delay the degradation of the polymeric matrix, leading to a decreased release of drug retained in this matrix [32,40].

We sought to identify which model would adequately explain the release kinetics of the encapsulated drug. The tests indicated, based on the correlation coefficient between the tested models shown in Table 2, that drug release is governed by the Higuchi model, which indicates release through diffusion. Thus, this model indicates that the environment surrounding the polymeric nanoparticle slowly penetrates the polymeric matrix based on PCL, an aliphatic polyester susceptible to hydrolytic degradation by breaking the ester bonds, dissolving it, and causing the diffusion of the drug RSV [21,42].

### 3.2. In Vitro Evaluation in Breast Cancer Cell Line

**Figure 4:**
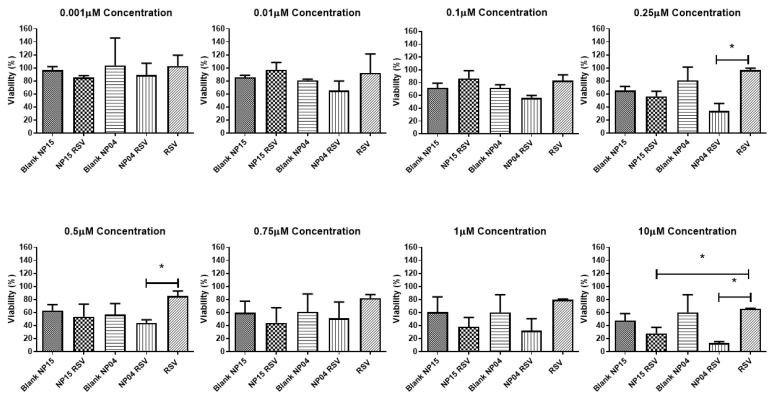
Cell viability in 4T1 cells of NP15 and NP04 formulations with and without RSV, and RSV in solution. Student’s *t*-test between NP15 RSV vs. RSV and NP04 RSV vs. RSV. * *p* < 0.05.

**Figure 5:**
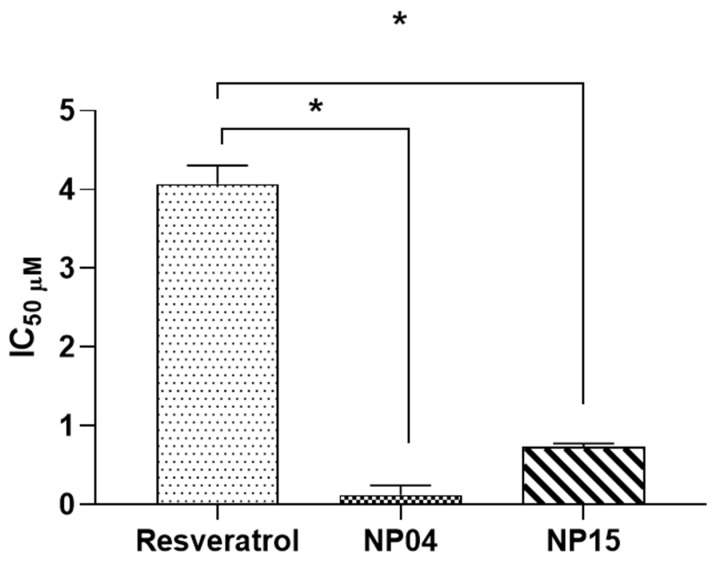
IC_50_ of free RSV, RSV-loaded formulations NP04 and NP15 with their respective standard deviations. Student’s *t*-test between NP15 RSV vs. RSV and NP04 RSV vs. RSV. * *p* < 0.05.

Herein, the cytotoxicity of the 4T1 breast cancer cell line was evaluated for blank NPs and RSV-loaded NPs. The results showed a significant reduction in cell viability caused by NPs loaded with RSV in almost all tested concentrations. NP04 and NP15 formulations showed greater cytotoxic activity compared to blank formulations and free drugs, 87.2% and 72.1%, respectively (Figure 4). A dose-dependent cytotoxicity was observed for both the nanoformulations and free RSV, but the highest activity was observed in NPs loaded with RSV. Encapsulation of RSV in polymeric NPs led to increased cytotoxicity compared to free RSV. Additionally, it was found that TPGS can impair cytotoxicity. Previous studies have shown that the encapsulation of RSV against various cell lines including DU-145, LNCaP, PC-3 [21], MCF-7 [25,40], and MDA-MB-231 [25] results in a significant reduction of the IC_50_ of RSV when it is encapsulated, also shown herein (Figure 5). Other works have also confirmed the antiproliferative activity of resveratrol (RSV) and its ability to induce apoptosis in 4T1 cells, resulting in cell cycle arrest in the synthesis phase (S) and a reduction in the number of cells in the G1/G0 phase [43]. Moreover, studies have indicated that resveratrol can inhibit colony formation in other breast cancer cell lines, such as MDA-MB-231, which is negative for the human estrogen receptor [44] and the MCF-7 cells [45].

The NP15 formulation contains TPGS, a compound that inhibits the regulation of anti-apoptotic genes and acts on mitochondria, leading to cell death. TPGS also inhibits P-glycoprotein, an efflux pump capable of expelling a large amount of antitumor drugs, potentially explaining its cytotoxic activity despite releasing less drug than the NP04 formulation [13,46].

**Figure 6:**
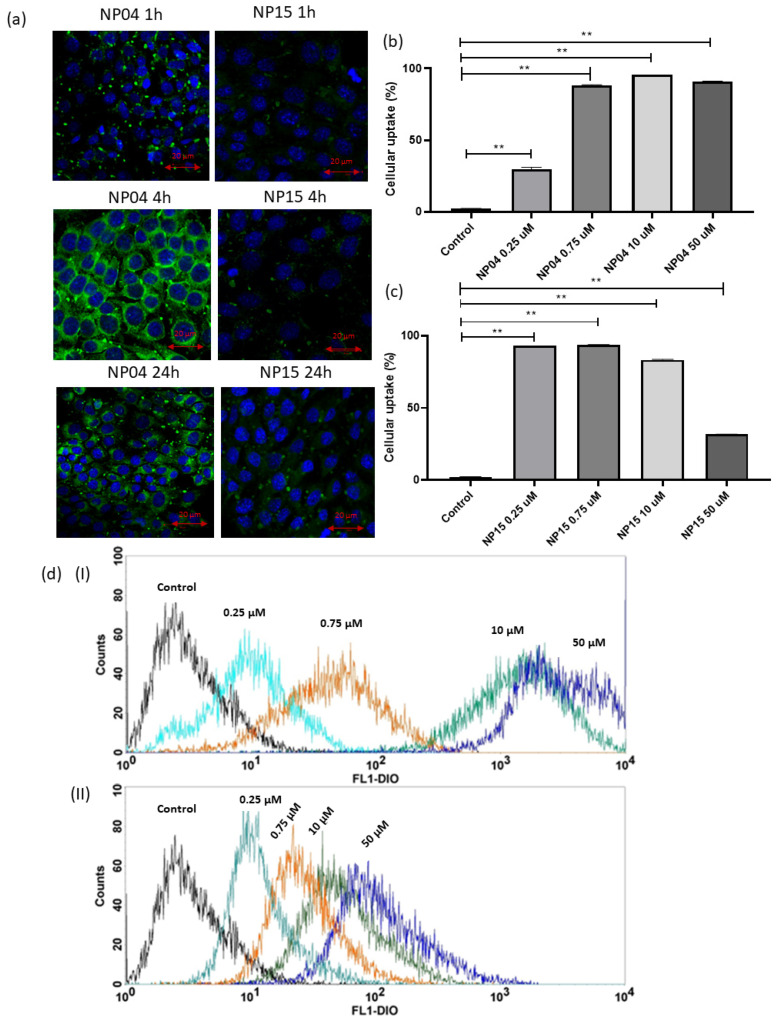
(**a**) Images by confocal microscopy of cellular uptake of nanoparticles in 4T1 cancer cells at treatment times of 1 h, 4 h, and 24 h, respectively, for NP04 and NP15 formulations. (**b**) Graphs showing the cellular uptake of the NP04 formulation with increasing concentrations. (**c**) Graphs showing the cellular uptake of the NP15 formulation with increasing concentrations. (**d**) Overall graphs of fluorescence vs. concentration for formulation NP04 (**I**) and for formulation NP15 (**II**). (**b**,**c**) One-way ANOVA test with Tukey post-hoc test ** *p* < 0.01.

In Figure 6a, the images of confocal microscopy demonstrated the cellular uptake of NPs NP04 and NP15 in 4T1 cells. It is possible to verify that the uptake of NPs by the cells increased over time until 4 h and decreased after 24 h. We also observed that the highest concentration of NPs is found in the cellular cytoplasm, where the release of the drug will occur in a more pronounced way. Previous works observed the concentration of TPGS-based polymeric NPs in the cytoplasm of MCF-7 and MDA-MB-231 breast cancer cells, and the same pattern of concentration of NPs in the cytoplasm and around the nucleus [21,25].

Observing the images of uptake by microscopy, it is clear that the NP04 formulation promoted a greater internalization of DIO-labeled NPs than NP15. In NP15, we assume that the lower cellular uptake is due to the presence of the PEG derivative, which is capable of leading to the formation of a steric and physical impediment that impairs the uptake of NPs from the formulation by the cells, corroborating the results obtained in the in vitro release and also in agreement with the results obtained in the cytotoxicity test [21].

#### Evaluation of Cell Uptake by Flow Cytometry

In order to quantify the internalization of DIO-labeled NPs, flow cytometry demonstrated that fluorescence varies according to concentrations. which is indicated by increased fluorescence of the DIO (Figure 6b–d). It was noticed that the NP04 formulation presents a greater internalization than the NP15, moreover, it is interesting that when comparing the internalization graphics, the NP04 formulation presents a direct correlation with the increase in concentration, while the NP15 formulation presents an inverse correlation. Furthermore, the flow cytometry results are corroborated by the confocal images (Figure 6a) and the MTT results (Figure 5), both favoring the NP04 formulation over NP15. While flow cytometry and confocal images prove a greater internalization of NP04, the MTT shows that the internalization leads to a more potent action against the breast cancer cells by the NP04 formulation, without TPGS.

Another point that can justify the better cell internalization behavior of NP4 formulation is the presence of the surfactant poloxamer 407, which is capable of causing an increase in the microviscosity of the cell membrane, allowing an improvement in endocytosis [25]. However, the presence of TPGS in NP15 formulation impairs cellular uptake.

### 3.3. In Vivo Evaluation in a Breast Cancer Xenograft Tumor Model

**Figure 7:**
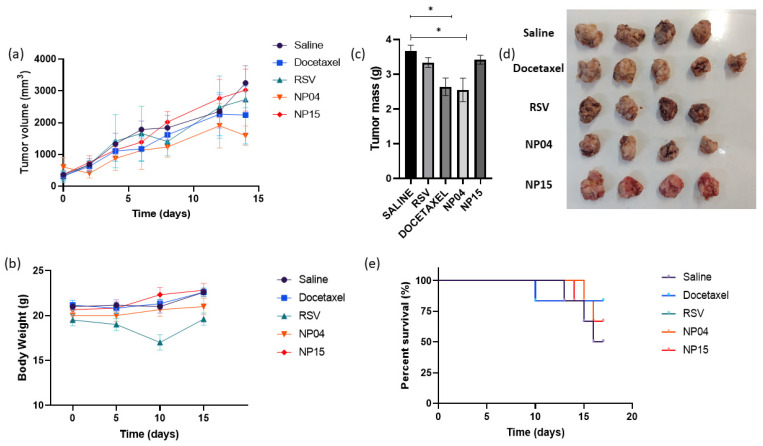
(**a**) Tumor growth graph of breast cancer-bearing mice treated with saline, docetaxel, free RSV, and NP4 and NP 15 formulations. (**b**) Body weight curve following administrations. (**c**) Tumoral mean weight from each group taken at the end of the study (* *p* < 0.05). (**d**) Image of tumors taken out of the mice from each group by the end of the study. (**e**) Percent survival of the animals throughout the study.

Subcutaneous 4T1 breast tumors were used to evaluate the in vivo therapeutic efficacy of the NP04 (without TPGS) and NP15 (with TPGS) nanoparticles compared to the antitumor drug docetaxel, free RSV, and the negative control saline group. The body weight of the mice remained constant within the different groups. The RSV group showed weight loss but later recovered (Figure 7b). Evaluating the survival of animals in each group, toxicity was observed in the docetaxel group, which led to the premature death of one of the mice, indicating that although it acted to reduce the tumor, it led to toxicity that was not observed in the other groups, as noted by the percent survival graph (Figure 7e) [47].

The growth of the tumor remained constant in all groups (Figure 7a). However, a significant increasing trend in tumor size for the saline group was evident. The NP04 formulation showed a significant reduction in the tumor growth rate (70.35 mm^3^/day) compared to the RSV (173.07 mm^3^/day), saline group (206.2 mm^3^/day), and NP15 group (189.4 mm^3^/day) contrary to previous findings that indicated inactivity of RSV against 4T1 cells [6], suggesting a superior activity compared to docetaxel. Upon evaluating the tumor mass of the groups after treatment completion (Figure 7d), the NP04 formulation (2.55 g ± 0.69) and docetaxel (2.64 g ± 0.56) showed significantly smaller tumor mass compared to the control saline group (3.67 g ± 0.34). On the other hand, the NP15 formulation (3.42 g ± 0.26) did not show a significant reduction in tumor mass (Figure 7c,d), in accordance with all the other in vitro results obtained herein, demonstrating that the NP04 formulation has greater activity against 4T1 cell line compared to the NP15 formulation, suggesting an interference of TPGS in the delivery of RSV from polymeric nanoparticles.

**Figure 8:**
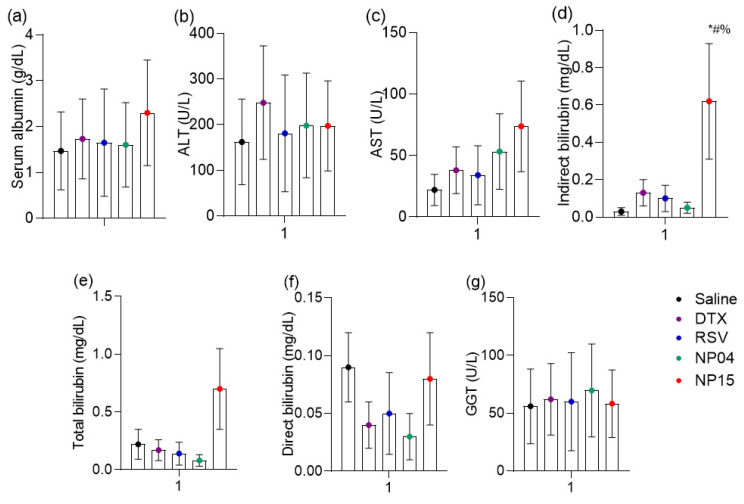
Evaluation of serum biochemical parameters after treatment with saline, docetaxel (dtx), resveratrol (RSV), and polymeric nanoparticles NP04 and NP15. (**a**) Albumin, (**b**) alanine aminotransferase (ALT), (**c**) aspartate aminotransferase (AST), (**d**) indirect bilirubin, (**e**) total bilirubin, (**f**) direct bilirubin, (**g**) gamma-glutamyl transferase (GGT). ANOVA with Bonferroni post-hoc, *p* < 0.05: * vs. salina; # vs. DTX; % vs. RSV.

In order to evaluate and compare the toxicity of the formulations, biochemical dosages were made, as shown in Figure 8. It was observed that the groups showed no difference in serum albumin (Figure 8a). This observation is consistent with the kinetics of albumin, which is produced in the liver and has an average life span in circulation of 20 days, presenting changes in serum levels more directed to subacute and chronic liver damage [48]. In order to perform more sensitive monitoring of liver toxicity, aminotransferases, also called transaminases, play a crucial role. ALT is a cytosolic enzyme and the increase in its activity in plasma indicates high specificity liver damage; AST, a mitochondrial enzyme, is also present in tissues such as skeletal and cardiac muscle. AST has slower-release kinetics and may indicate later liver changes that exceed 48 h [49]. In this sense, the results revealed that the ALT (Figure 8b) showed no difference in activity depending on the treatments as well as AST (Figure 8c).

Liver toxicity data are confirmed with the determination of bilirubin, which is produced in the spleen as a product of the degradation of the heme group of senescent red blood cells. After heme oxidation processes, bilirubin is formed which is transferred to the liver bound to albumin. In the liver, bilirubin is conjugated to glucuronic acid by the action of the enzyme UDP-glucuronosyltransferase, through phase II metabolism reactions, which makes bilirubin conjugated, or directly water-soluble, allowing its fecal elimination via gallbladders. Increased serum levels of indirect or unconjugated bilirubin are early markers with high specificity for hepatocyte damage and consequent alteration of conjugation [50]. The present study showed increased levels of indirect bilirubin in the NP15-treated group compared to the saline-treated control with the docetaxel-treated group and also with free resveratrol (Figure 8d).

Liver changes may be toxic or obstructive. In obstructive changes, bilirubin undergoes conjugation in hepatocytes, but cannot reach the gallbladder, causing this conjugated or direct bilirubin to accumulate in the liver tissue and be drained by the bloodstream. Thus, obstructive diseases occur with an increase in direct bilirubin with or without an increase in total bilirubin, the total being the sum of direct and indirect concentrations. One way to confirm obstruction is by evaluating the marker gamma-glutamyl transferase (GGT); this enzyme has its activity increased in plasma in virtually all hepatic obstructive processes [51]. The total bilirubin, direct bilirubin, and GGT data together are not suggestive of hepatic obstruction. Thus, the slight increase in indirect bilirubin is suggestive of the competition of NP-15 with bilirubin by the catalytic site of the metabolizing enzyme, as with a variety of drugs, such as rifampicin [50].

## 4. Conclusions

The purpose of this work was the development and characterization of PCL-based nanoparticles with the aim of encapsulating drug resveratrol, in addition to studying how TPGS could influence the physicochemical characteristics of nanoparticles, as well as their effect on breast cancer, in vitro and in vivo. The nanoparticles were produced presenting nanometric size with satisfactory polydispersion index. Furthermore, we evidenced the role of TPGS as an adjuvant in the formation of nanoparticles. The high efficiency of resveratrol encapsulation in polycaprolactone-based nanoparticles was also noted. The cytotoxicity results were promising, indicating a potent action of the encapsulated resveratrol against the breast cancer cell line, particularly for the formulation without TPGS. The in vitro release, cell uptake, and cytotoxicity studies indicated that TPGS led to a slower and reduced release, thus decreasing its cytotoxicity, which correlates with the decrease in cellular uptake also seen for the formulation with this TPGS. Finally, the in vivo antitumoral effect findings showed that the NP04 formulation showed a better control on the rate of tumor growth and proved to be non-hepatotoxic, demonstrating to be more effective than not only the free drug but also than docetaxel and the NP15 formulation, corroborating all the other results, which indicate a better action of the formulation without TPGS compared to the one that was functionalized with such material.

## Figures and Tables

**Table 1:** Physicochemical characterization of the formulations in relation to particle size, polydispersity index (PDI), zeta potential, and encapsulation efficiency (EE).

Formulation	Composition Drug (mg)/Acetone (mL)/TPGS(%)	Size (nm) ± SD	PDI ± SD	Zeta Potential (mV) ± SD	EE (%) ± SD
**NP 01 RSV**	10/10/0	138.8 ± 5.0	0.19 ± 0.01	−4.95 ± 0.47	98.02 ± 0.32
**NP 02 RSV**	01/10/0	146.2 ± 0.9	0.11 ± 0.01	−2.53 ± 0.22	95.88 ± 0.23
**NP 03 RSV**	05/10/0	149.0 ± 0.7	0.13 ± 0.01	−2.56 ± 0.18	97.03 ± 0.32
**NP04 RSV**	10/05/0	138.1 ± 1.8	0.18 ± 0.01	−2.42 ± 0.56	98.21 ± 0.87
**NP 05 RSV**	05/05/0	145.9 ± 2.5	0.13 ± 0.01	−2.66 ± 0.51	97.76 ± 0.24
**NP 06 RSV**	01/05/0	152.5 ± 0.5	0.10 ± 0.01	−1.87 ± 0.23	96.96 ± 0.56
**NP 12 RSV**	10/10/0.001	132.2 ± 0.8	0.19 ± 0.01	−3.12 ± 0.35	96.80 ± 0.15
**NP 13 RSV**	10/10/0.005	131.3 ± 0.8	0.19 ± 0.01	−2.34 ± 0.38	92.48 ± 0.45
**NP 14 RSV**	10/10/0.010	127.3 ± 1.6	0.17 ± 0.01	−2.16 ± 0.32	85.16 ± 3.53
**NP15 RSV**	10/10/0.015	127.5 ± 3.1	0.19 ± 0.01	−2.91 ± 0.90	98.40 ± 0.01
**Mean**		138.6 ± 9.2	0.16 ± 0.04	−2.75 ± 0.85	96.60 ± 1.84

**Table 2:** Determination coefficient values of the release models from nanoparticles NP04 and NP15.

				R^2^
	Zero Order	1st Order	Higuchi	Korsmeyer-Peppas
**NP04**	0.21	0.73	0.89	0.78
**NP15**	0.67	0.85	0.95	0.87

## Data Availability

The data presented in this study are available on request from the corresponding author.

## Abbreviations

DAPI	4′,6-diamidino-2-phenylindole
DiO	3,3′-dioctadecyloxacarbocyanine perchlorate
DMSO	Dimethyl sulfoxide
DSC	Differential Scanning Calorimetry
EE	Encapsulation efficiency
EPR	Enhanced permeability and retention
FTIR	Fourier Transform Infrared Spectroscopy
HER2	Epidermal growth factor 2
IGF-2	Insulin-like growth factor 2
MTT	3-[4,5-dimethyl-thiazol-2-yl]-2,5-diphenyltetrazolium bromide
NPs	Polymeric nanoparticles
PBS	Phosphate-buffered saline
PCL	Poly-ε-caprolactone
PDI	Polydispersity index
PEG	Polyethylene glycol
RSV	Resveratrol
SEM	Scanning electron microscopy
TPGS	D-α-tocopheryl polyethylene glycol 1000 succinate
